# Recent advances in porous graphitic frameworks: a critical mini-review

**DOI:** 10.1039/d5ra07821k

**Published:** 2025-12-16

**Authors:** Minh Kim Nguyen, Ha Huu Do, Nguyen Tien Tran, Young-Chul Lee

**Affiliations:** a Department of Nanoscience and Technology Convergence, Gachon University Gyeonggi-do 13120 South Korea; b NTT Hi-Tech Institute, Nguyen Tat Thanh University Ho Chi Minh City Vietnam; c Nguyen Tat Thanh University Center for Hi-Tech Development Saigon Hi-Tech Park Ho Chi Minh City Vietnam; d Center for Advanced Chemistry, Institute of Research and Development, Duy Tan University Da Nang 550000 Vietnam trannguyentien@duytan.edu.vn; e Faculty of Natural Sciences, Duy Tan University Da Nang 550000 Vietnam; f Department of Bionanotechnology, Gachon University Gyeonggi-do 13120 South Korea dreamdbs@gachon.ac.kr

## Abstract

Porous graphitic frameworks (PGFs) represent a rapidly maturing class of sp^2^-carbon architectures that reconcile the traditionally antagonistic requirements of very-high surface area, hierarchical mass-transport pathways, and metallic-level electronic conductivity. This mini-review critically surveys the latest advances in their bottom-up construction, from hard-templated and chemical-vapor-deposited networks to emerging inside-out activation–graphitization protocols. This review also correlates these synthetic routes with key structural attributes, surface area, pore-size distribution, and graphitic domain continuity, and elucidates their role in providing exceptional electrical, thermal, and mechanical performance metrics. Application case studies include high-rate supercapacitors, single-atom electrocatalysts, Ångström-precision gas-separation membranes, chemiresistive sensors, and high-capacity adsorbents. Data-driven process control, molten-salt-mediated doping, and additive manufacturing are expected to deliver application-specific PGFs at scale, while advanced functionalization strategies decouple active-site chemistry from bulk conductivity. Collectively, the combination of sustainable synthesis, tunable nanochemistry, and wide applications makes PGFs an effective material platform for next-generation energy, environmental, and electronic technologies.

## Abstract 2

This article focuses on the most recent synthetic advances of PGFs and summarizes structural characterization methods, emergent behavior, and application horizons.

## Graphical abstract

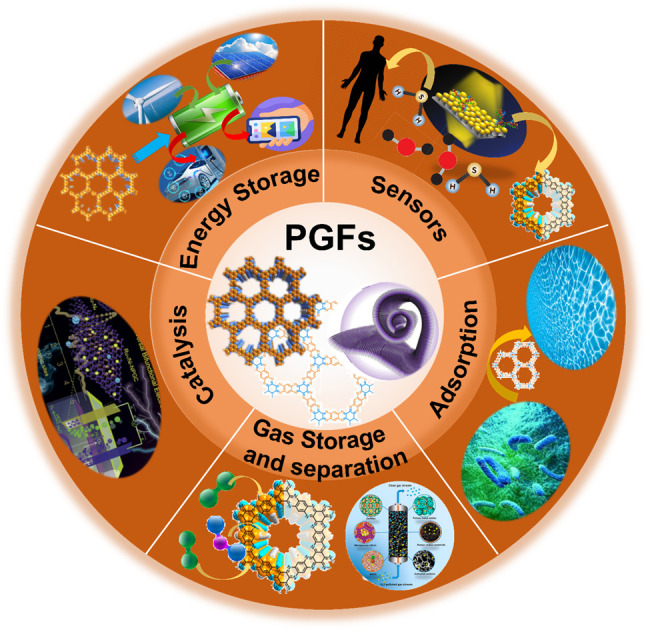

## 1. Introduction

Porous graphitic architectures (PGFs) are a new class of carbonaceous materials, which possess a high surface area, the tunable porosity of conventional activated carbons with long range π conjugation, and the crystallographic order of graphitic domains. This combined structural motif directly addresses the long-standing bottlenecks in electrochemical and catalytic technologies, namely, slow charge transport and mass transfer, by providing continuous sp^2^ carbon highways through an open-pore network. The rapidly growing volume of the literature on 3D porous graphene architectures for energy storage reflects the growing strategic importance of this design paradigm.^1^

At the molecular level, PGFs are built from perfectly annulated aromatic backbones, in which rigid ladder-like linkages fix neighboring benzene or heteroaromatic units to planar sheets. Periodic voids are incorporated either by intrinsic ring fusion or sacrificial templating. The demonstration of dynamic covalent C=N exchange in pyrazine rings by Li *et al.* (2020) was a breakthrough, allowing for crystalline PGF, providing a template for the attainment of in-plane order under relatively mild hydrothermal conditions, and the discovery of reversible bond formation as an essential function in error correction during framework development.^2^ Follow-up ladder polymer strategies have been developed based on this concept to create conjugated porous networks with redox-active backbones that can be systematically optimized for battery electrodes and other platforms for charge storage.^3^

The resulting materials possess a set of properties that are difficult to obtain simultaneously using conventional carbons. Electrical conductivities above 10 S cm^−1^, Brunauer–Emmett–Teller (BET) surface areas always greater than 1 000 m^2^ g^−1^, and excellent thermal and chemical stability due to graphitic bonding. Recent studies have emphasized how these properties translate into functional advantages. For instance, metal–organic frameworks (MOF)-derived PGFs with spinel NiFe_2_O_4_ nanoparticle decorations exhibit remarkably faster perchlorate degradation rates by synergistically bridging conductive carbon highways and rich catalytic interfaces,^4^ while Kr adsorption research now allows quantitative edge plane density mapping of graphitic carbons, giving a metrology for defect chemistry correlation with adsorption or electrocatalytic activity.^5^

**Fig. 1:**
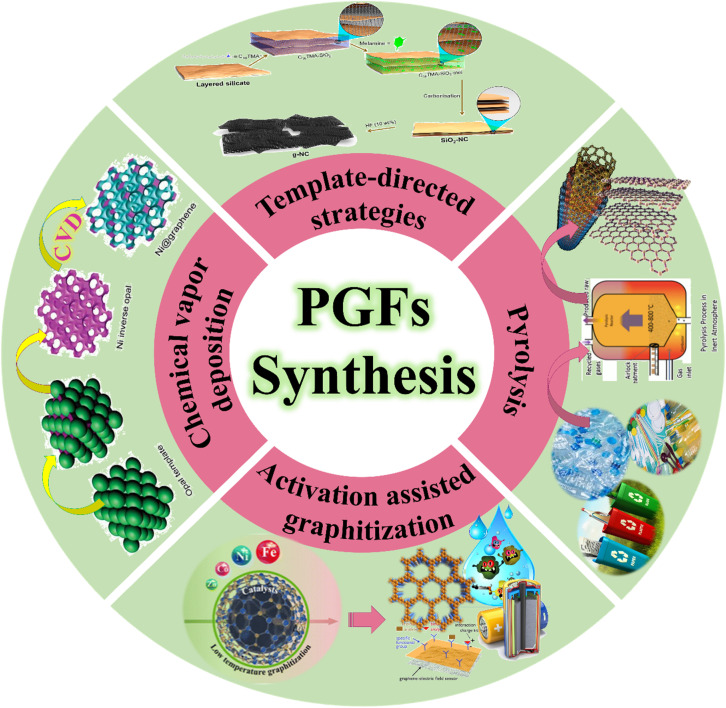
PGFs synthesis techniques.

Despite these developments, significant knowledge gaps remain in the literature. However, scalable synthesis routes that balance crystallinity, heteroatom incorporation, and hierarchical pore development remain elusive. *In situ* characterization techniques capable of resolving ion or molecule transport within sub-nanometer graphitic channels are only beginning to be developed. This mini-review focuses on the most recent synthetic advances (Fig. 1). Besides, it also summarizes structural characterization methods, emergent behavior, and application horizons of PGFs toward distilling structure–function relationships to guide the rational design of future graphitic porous materials.

## 2. Synthesis methods

Porous graphitic frameworks can be synthesized *via* several conceptual routes that differ in how graphitic domains nucleate, how pore geometry is templated, and how heteroatom chemistry is incorporated. Accordingly, three different synthesis approaches groups can be mentioned. Accordingly, soft-templated or supramolecular-templated routes, in which micellar, block-copolymer, or hydrogen-bonded assemblies are used to pre-organize carbon or nitrogen-rich precursors into meso-structured domains that are subsequently carbonized.^6–9^ Hard-templated and CVD-assisted routes, where rigid silica, polymer, or 3D graphene scaffolds support the formation of hierarchical pore networks coupled with graphitization.^10–12^ And MOF/COF-derived or salt-assisted pyrolysis strategies, in which reticular frameworks or supramolecular mixed matrices are transformed into N-doped graphitic carbons with atomically dispersed metals and well-defined micro/mesopores.^13,14^

Hard-templating (*e.g.*, silica, layered silicates, MgO) provides the high geometric precision and often forms ordered or pseudo-ordered mesopores after template removal, as demonstrated in recent studies producing graphitic nitrogen-enriched carbons using exfoliable silicate platelets as sacrificial scaffolds.^6^ Conversely, soft-templates, such as block-copolymers, supramolecular micelles, and ionic surfactant assemblies, enable large-area meso-structure formation without corrosive etching, as shown in mesoporous graphitic carbons derived from Pluronic-directed assemblies.^15^ CVD approaches provide atomic-level control of sp^2^ domain and allow heteroatom integration, consistent with reports using methane decomposition on MgO microspheres to yield double-layer graphitic membrane which possessed approximated BET of 1500 m^2^ g^−1^.^8^ Finally, pyrolysis-based graphitization of MOFs, conjugated polymers, biomass, and supramolecular matrices has become the flexible route for scalable PGF production, especially when combined with catalytic graphitization (Ni, Fe, Co) or activation (K-based, molten salts).^16–18^ These routes have recently achieved hierarchically porous carbons with controllable micro/mesopore ratios, graphitization indices, and electrical conductivities reaching 10 S cm^−1^.^9,13,19^

Recent reports on nitrogen-doped porous graphitic carbon prepared by layered double hydroxide templating,^6^ conjugated-polymer-derived 3D porous graphitic carbons,^7^ and 3D porous graphene/carbon nanocage architectures^8^ represent the first two families, whereas Ni-MOF-derived Ru–N_4_ single-atom catalysts,^13^ ZIF-derived Zn single-atom-embedded carbon molecular sieves,^9^ and NaCl-assisted ZIF-8 carbonization towards porous N-doped carbons hosting precious-metal single atoms^19^ illustrate the third family. In parallel, g-C_3_N_4_-type porous graphitic frameworks (*e.g.*, mesoporous g-C_3_N_4_ nanosheets and heterostructures) are classified as nitrogen-rich analogues that share similar synthetic soft-templating, defect/dopant engineering, and hierarchical morphology control.^20–23^ However, they operate primarily as semiconducting photocatalysts rather than purely conductive carbons.^10,24–26^

### 2.1. Template-directed strategies

**Fig. 2:**
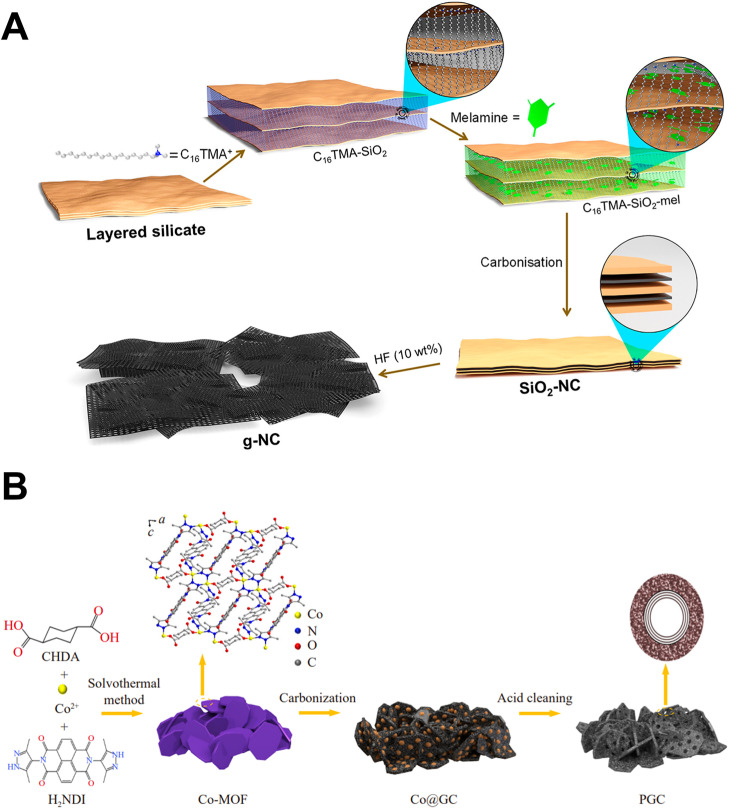
(A) Synthesis scheme for g-NC using hard templating.^6^ Reuse permission from Doustkhah *et al.* (2023)^6^ by American Chemical Society. (B) Synthesis of porous graphitic carbons from Co-MOF precursors.^29^ Reuse permission from Chu *et al.* (2024)^29^ by Springer Nature, under a Creative Commons Attribution 4.0 International License (https://creativecommons.org/licenses/by/4.0/).

Hard templating is the most structurally deterministic method for obtaining porous graphitic frameworks (PGFs). The recent work of Doustkhah *et al.* (2023) used exfoliable 2D layered silicate platelets as sacrificial templates.^6^ Pyrolysis of a melamine–cetyltrimethyl–ammonium composite and carbonization–activation process of chitosan/polyethylene glycol (PEG) blend or melamine–resorcinol–formaldehyde (M–R–F) co-polymeric microspheres could provide nitrogen-rich graphitic carbons with a micro/mesopore-architectures that replicated the interlayer gallery.^6,27,28^ Density functional analysis confirmed graphitic N as the thermodynamic sink, accounting for the material's 90% capacitance retention up to 10 000 cycles (Fig. 2A).^6^ In a self-templated complementary counterpart, nano CaCO_3_ was utilized to form pores and serve as an *in situ* CO_2_ source to prevent sheet restacking. The resulting CaCO_3_/coffee ground hybrid had a discharge of 760 mA h g^−1^ after 100 LIB cycles, illustrating a pathway for using transient inorganic domains to control porosity and heteroatom doping simultaneously.^1^ Despite this success, mineral template removal (thermal volatilization or acid leaching) remains energy-intensive and limits scalability. Therefore, the field is moving toward bio-templated or melt-salt approaches that integrate template decomposition with graphitization.

Besides, soft-template strategies use surfactant assemblies, block-copolymer micelles, supramolecular hydrogen-bonded networks, or ionic liquid–polymer co-assemblies—are capable of producing mesoporous carbons with ordered or semi-ordered pore lattices without the corrosive template etching required in hard templating. Recent reviews on mesoporous graphitic/non-graphitic carbons^11,12^ highlight how Pluronic F127, P123, CTAB, and related amphiphiles can direct precursor polymerization prior to carbonization, yielding tunable 2–50 nm mesopores with high pore volumes and uniform distributions. In addition, soft-templated graphitic carbon nitride (g-C_3_N_4_) systems, particularly mesoporous or doped g-C_3_N_4_, follow analogous supramolecular-assembly principles and are widely employed in photoactive or redox applications.^10,20–26^

Hard-templated PGFs, such as silica, layered silicates, CaCO_3_, and MgO, often exhibit high graphitic ordering and well-defined mesopore channels after template removal, as confirmed through Raman *I*_D_/*I*_G_ ratios, XRD, and TEM analyses. They produce pore geometries that directly replicate the template lattice. Representative examples include exfoliable silicate-templated nitrogen-rich graphitic carbons^6^ and MgO-derived graphenic nanocages.^8^ By contrast, soft templates, while generally less crystalline, typically generate ordered mesopores and offer enhanced dopant homogeneity and better control over mesopore dimensions due to the uniformity of micellar domains. However, they lack rigid anchoring points during carbonization. Thereby, these aspects make them advantageous for scalable synthesis and facile doping, yet more susceptible to framework shrinkage. Soft-templated mesoporous carbons and mesoporous g-C_3_N_4_ demonstrate these trade-offs, and their common structural motifs have now been explicitly discussed using recent literature.^14,21,22^

### 2.2. Chemical vapor deposition on 3D scaffolds

Chemical vapor deposition (CVD) offers atomic-level control of the graphitic layer growth and separates carbon nucleation from pore formation. Zhu *et al.* (2019) demonstrated a fluidized bed CVD process where CH_4_ decomposes on MgO microspheres at 950 °C to form a conformal double-layer graphene skin that preserves the sacrificial template's nest like microporosity. After MgO removal, the scaffold exhibited a BET surface area of 1460 m^2^ g^−1^ and tolerated 1560 mA h g^−1^ in LIB anodes.^1,8^ The gas phase nature of CVD enables seamless incorporation of heteroatom dopants (*e.g.*, B_2_H_6_, NH_3_) or catalytic nanoparticles onto growth but is constrained by throughput by residence time and precursor consumption, inefficiencies of limitations addressed by microwave plasma and roll to roll aerosol CVD reactors.

### 2.3. Pyrolysis of pre-organized organic precursors

The direct carbonization of covalent organic frameworks, MOFs, and biomass produces PGFs whose pore structures are imprinted in the precursor. Chu *et al.* carbonized a triply interpenetrated Co-MOF at 1300 °C to produce PGC 1300, featuring a high graphitization index and interconnected 1–5 nm mesopores. The anode delivered 128.5 mA h g^−1^ at 3.2 A g^−1^ in lithium-ion capacitors and retained 91.6% capacity after 10 000 cycles (Fig. 2B).^29^ Parallel biomass-derived routes leverage the inherent hierarchical routes. Precursors of peanut shell and pumpkin skin, *via* two stage calcination, yield graphitic carbons with surface area >2 000 m^2^ g^−1^ and oxygen functionalities facilitating K-ion adsorption.^30–32^ Significant bottlenecks include precursor heterogeneity and the need for high temperatures (>1200 °C) for sp^2^ ordering. Catalytic graphitization using Fe, Ni, or Co salts is more commonly used to minimize energy footprint (Table 1).

**Table 1:** The various synthesis methods, properties, and the applications of PGFs materials

Method	Precursors	Conditions	Product	Applications	Ref.
Hard templating	Layered silicate, melamine and cetyltrimethyl ammonium (C16TMA)	Heated at 800 °C	g-NC (C_6.3_H_3.6_N_1_O_1.2_)	Electrocatalytic, supercapacitance	6
Hard templating	Coffee ground, calcium carbonate and iron nitrate	Carbonized at 750 °C	NC3320	Electrochemical	1
CVD	CH_4_ and MgO catalyst	950 °C in fluidized bed reactor	S/PHG-5	Electrochemical	8
Pyrolysis	Co-MOF	Pyrolysis at 900–1500 °C	PGC-*x*	Electrochemical, lithium-ion capacitor	29
Pyrolysis	Pumpkin skin powder	Pyrolysis at 900 °C	GHPC	Electrochemical, supercapacitors	30
Pyrolysis	K_2_CO_3_, g-C_3_N_4_	Pyrolysis at 700–900 °C	ONPC-900	Electrochemical, supercapacitors	33
Pyrolysis- flash Joule heating	Sawdust, bamboo, and rice straw	Vacuum desiccator ∼0.6 psi, 200 V, 50 Hz	FG	Catalyzed hydrogenation, photothermal conversion	31
*In situ* activation–graphitization	Potassium citrate and iron citrate	Carbonized at 700–800 °C	HPGC-*T*	Energy storage, supercapacitor	34
Chemical activation–graphitization	PANi hydrogel	Carbonized at 400–900 °C	3D HPG	Energy storage, supercapacitor	7
Flash Joule heating	Graphene oxide	Heated at 3 000 K (∼300 K min^−1^)	NP@rGO aerogel	—	35
Molten salt-assisted	ZIF-8 powder and NaCl	Heated at 950 °C	PM_1_/N–C_Pores_	Electrocatalytic	19

### 2.4. Activation-assisted graphitization

Physical or chemical activation increases the number of micropores and causes defects but traditionally at the cost of conductivity. Zhao and Zhang solved this problem using an *in situ* activation–graphitization process. Atomically dispersed K (activator) and Fe (graphitization catalyst) in precursors of potassium/ferric citrate lead to “inside out” growth of pores along with ordering of the lattice to give hierarchical PGF with 322 F g^−1^ and 101% capacitance retention after 15 000 cycles.^34^ Environmentally friendly activating agents such as KHCO_3_ and Na_2_CO_3_, and molten salt media now replace corrosive KOH/ZnCl_2_, suppressing equipment corrosion while enabling closed loop recovery of salt. However, precise control of the one-step activation pore size distribution is still inaccessible, stimulating *operando* small-angle X-ray scattering research to correlate gas and pore evolution in real time.

### 2.5. Advantages and disadvantages across various aspects

#### 2.5.1. Energy cost

Hard-templated carbons and MOF-derived PGFs typically require 800–1500 °C to achieve graphitic ordering, as demonstrated in recent high-temperature carbonization of Co-MOF precursors.^29^ CVD also operates in the 900–1100 °C regime, consuming additional energy within gas-phase reactors, thereby increasing overall operational cost.^8^ In contrast, molten-salt graphitization has recently achieved graphitic ordering at significantly lower temperatures (∼900 °C) due to ionic confinement and enhanced heat transfer,^19^ while ultrafast flash Joule heating (FJH) reaches over 3 000 K for below 1 s, producing graphitized carbons in kilogram-scale at only 2–3 kWh kg^−1^.^31^

#### 2.5.2. Scalability

Hard-templating remains the least scalable technique because both the templating and template removal steps impose intrinsic throughput limitations.^1,6,29,32^ Soft-templating routes, which base on amphiphilic micelles or block copolymers, provide better scalability, although their reproducibility can be influenced by precursor and micelle stability.^10–12,24–26^ CVD provides excellent crystallinity but is still limited in reactor size.^8,10–12^ MOF-derived carbons offer moderate scalability, but precursor costs interfere large-scale adoption.^13,14,16–18,29^ Recent advances in molten-salt and FJH processes demonstrate the highest scalability potential, particularly for biomass-derived PGFs, since both molten salts and FJH processes enable high-volume, low-cost throughput.^19,31^

#### 2.5.3. Structural control

Hard-templated PGFs exhibit the high pore precision, as the sacrificial template allows determined pore architectures.^6^ Soft-templating generates uniform mesopores *via* micellar assemblies but provides less control over micropores.^10–12,24–26^ CVD enables atomic-level crystalline control, although internal porosity is difficult to regulate without additional scaffolds.^6–9^ MOF-derived PGFs offer well-defined microporous frameworks but frequently show collapses, resulting in heterogeneous porosity.^16–18^ Molten-salt routes provide excellent micropore sculpting and dopant stabilization, as molten salt ions keep structural development and prevent collapse.^19^ In contrast, FJH provides rapid graphitization. However, the ultrafast heating (<1 s) leads to less predictable pore formation despite high crystallinity.^31^

### 2.6. Emerging PGF synthesis strategies and perspectives

Emerging inside-out activation methodologies (*e.g.*, K/Fe-mediated hierarchical graphitization) have produced materials with high surface area (>3 000 m^2^ g^−1^) while maintaining conductivity.^34^ Molten-salt-mediated graphitization has enabled the formation of single-atom-stabilized porous graphitic carbons, such as Ru–N_4_ systems with high electrochemical performance.^19^ Flash Joule heating (FJH) has enabled ultrafast (<1 s) graphitization of biomass and polymers, producing high-SSA carbons at a lower energy cost than conventional thermal routes.^31,35^

In general, despite the advantages, these methods also have various disadvantages that need to be addressed. For instance, hard-templated PGFs remain difficult to scale due to multi-step template removal, energy-intensive calcination, and the high cost and waste generation associated with etching agents.^6,8^ CVD methods, though capable of producing highly crystalline sp^2^ frameworks, are limited by low throughput, precursor inefficiency, and reactor retention time limitations, as emphasized in fluidized-bed graphene growth studies. Conversely, MOF-/ZIF-derived graphitic carbons demonstrate promising scalability but face precursor heterogeneity and high-temperature requirements.^9,13,16–18^ Emerging molten-salt and flash Joule heating routes offer increased scalability but are still challenged by batch size limitations, uniform heat distribution, and trade-offs between graphitization and dopant retention.^19,35^ These challenges of the technical constraints limit industrial implementation of PGFs.

It can be emphasized that although diverse PGF synthesis platforms, such as hard/soft templating, CVD, MOF/COF pyrolysis, molten-salt graphitization, flash Joule heating, have made significant progress, there is no generalized and standardized methodology achieving simultaneous scalability, high graphitization, precise pore control, and sustainable processing. Hard templates remain the most structurally deterministic but are energy- and waste-intensive.^6,8^ Soft templates excel in scalability but struggle with high-temperature graphitization.^11^ MOF-/ZIF-derived PGFs provide chemically rich frameworks but face precursor cost and structural heterogeneity issues.^9,13^ Therefore, hybridized, multi-modal synthesis approaches are the most promising future direction.

Collectively, the template-directed assembly offers unmatched geometric precision, CVD offers crystallinity, pyrolysis provides chemical variability, and activation offers an ultrahigh surface area. Future synthetic paradigms will likely hybridize these modalities, for example, template-free salt activation with subsequent low-pressure CVD healing, to realize PGFs that optimize graphitic continuity with multiscale porosity while satisfying life-cycle sustainability metrics.

## 3. Structural characteristics

The formation of PGFs is typically validated by different essential characterization techniques, such as Raman and XRD spectroscopy for disorder or graphitic domain size (*I*_D_/*I*_G_ ratios, D-band width),^36,37^ pair distribution function (PDF) analysis for turbostratic stacking and crystallite size,^14^ N_2_ adsorption/desorption for BET surface areas or pore-size distribution in both crystalline and disordered carbonaceous frameworks,^36^ TEM or HRTEM for direct imaging of graphitic fringes, and XPS for heteroatom configurations (N-pyridinic, N-graphitic, *etc.*). This toolbox was informed by recent structural-characterization review,^14^ providing various useful characterization outputs and synthetic strategy.

### 3.1. High surface area

**Fig. 3:**
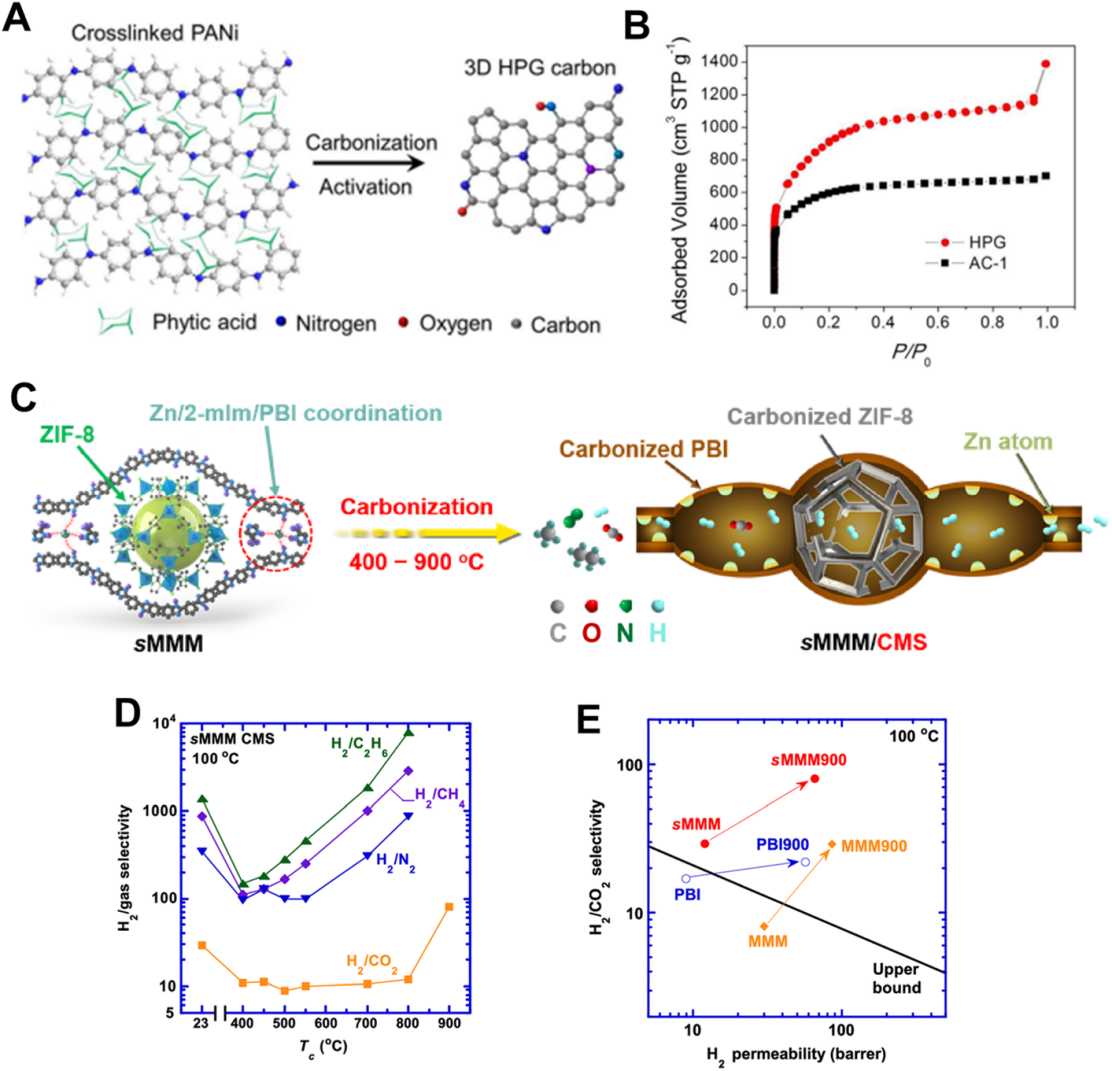
(A) Transformation of phytic-acid-crosslinked PANi (left) into doped graphene-like carbon sheets.^7^ (B) N_2_ adsorption/desorption isotherm of HPG carbon, clearly showing a hierarchically porous structure.^7^ Reuse permission from To *et al.* (2015)^7^ by American Chemical Society. (C) Schematic illustration of the carbonization of a supramolecular mixed-matrix material (sMMM) to form CMS materials containing polymodal free volumes from 400 to 900 °C.^9^ Reuse permission from Hu *et al.* (2024)^9^ by Nature, under a Creative Commons Attribution 4.0 International License (https://creativecommons.org/licenses/by/4.0/). (D) H_2_/gas selectivity at 100 °C showcasing the flexible tunability of separation properties by varying carbonization temperature (*T*_c_).^35^ Reuse permission from Xia *et al.* (2024)^35^ by American Chemical Society, under a Creative Commons Attribution 4.0 International License (https://creativecommons.org/licenses/by/4.0/). (E) Superior H_2_/CO_2_ separation properties of sMMM900 (E).^9^ Reuse permission from Hu *et al.* (2024)^9^ by Nature, under a Creative Commons Attribution 4.0 International License (https://creativecommons.org/licenses/by/4.0/).

The defining textural attribute of PGFs is their ability to sustain Brunauer–Emmett–Teller (BET) surface areas that rival, and in some cases exceed, those of conventional activated carbon, while retaining an electrically percolating sp^2^ lattice. Recent examples underscore the effectiveness of synchronous activation–graphitization chemistry.^7,38^ Singh *et al.* (2024) reported KOH/Fe-catalyzed carbothermic conversion of lignocellulosic precursors that produced nanoporous carbon–ceria composites with tunable SSA up to 1.997 m^2^ g^−1^ without compromising graphitic order.^38^ Higher values can be achieved when chemical activation is coupled with rigid conjugated polymer templates. To *et al.* (2015) achieved 4.073 m^2^ g^−1^ in a 3D hierarchically porous graphitic (HPG) carbon prepared from a phytic-acid-cross-linked polyaniline molecular framework, demonstrating that graphitization can proceed at 800 °C provided that the aromatic backbone is topologically locked prior to carbonization (Fig. 3A and B).^7^ Such ultrahigh-area PGFs mitigate the classical tradeoff between capacitance and rate capability in electrochemical double-layer capacitors by maximizing the ion-accessible area without introducing insulating amorphous carbon.

### 3.2. Tunable pore size

Beyond the magnitude, the distribution and connectivity of pores dictate the mass-transport phenomena. The carbonization of mixed-matrix precursors containing amorphous and crystalline ZIF-8 has emerged as a powerful route to achieve polymodal porosity. Hu *et al.* (2024) showed that the *in situ* dispersion of single Zn atoms within a carbon molecular-sieve membrane allows carbonization temperature (Fig. 3C) to dial the ultra-micro pore cutoff from 2.1 Å to 4 Å, enabling H_2_/CO_2_ selectivity up to 130 and H_2_/CH_4_ selectivity of 2.900. These values eclipse Robeson's upper bound for polymeric membranes (Fig. 3D and E).^9^ Complementary Monte-Carlo simulations of Kr adsorption in slit pores corroborate that Ångström-level aperture control can be extracted from noble gas sorption isotherms, providing rapid metrology for the pore-size profiling of graphitic carbon.^36^ Hierarchical design principles combine sacrificial hard templates (*e.g.*, MgO microspheres) for macroporosity with *in situ* catalytic activation to generate concentric micro- and mesopores, yielding surface-to-volume ratios that are optimized for both diffusion- and adsorption-limited applications.

### 3.3. Graphitic domains

The continuity and size of the sp^2^ domains govern charge transport, thermal stability, and chemical robustness. Quantitative Raman metrics, principally the *I*_D_/*I*_G_ ratio and D-band full width at half-maximum, provide a proxy for the in-plane crystallite size (*L*_a_) *via* the Tuinstra–Koenig formalism. A comparative study of 20 nanoporous carbons demonstrated that smaller graphene-like domains (lower *I*_D_/*I*_G_) correlated with enhanced supercapacitor capacitance, linking electronic disorder to ion-sieving efficiency.^37^ In hierarchically porous Zn-embedded carbons, the progressive drop in *I*_D_/*I*_G_ from 1.12 to 0.86 as carbonization temperature increased from 500 °C to 900 °C paralleled the emergence of a (002) reflection at 44°, confirming turbostratic stacking of graphitic layers.^9^ The total -scattering pair-distribution-function analyses further revealed that the nanocrystalline domains extend 2–4 nm and are intergrown with amorphous regions. This mixed ordering reconciles high conductivity (>10 S cm^−1^) with the defect sites necessary for catalytic functionality.^14^ Finally, edge-plane density, quantified *via* Kr adsorption differentials, has emerged as a critical descriptor for redox catalysis, offering an handle to balance basal-plane transport with edge-site reactivity in next-generation PGFs.^39^

### 3.4. Trade-offs, structure–property gaps, and perspectives

While several studies claim simultaneous optimization of both properties, recent Raman-based disorder analyses reveal that increased ultra-micro porosity often correlates with reduced sp^2^ domain continuity, leading to higher *I*_D_/*I*_G_ ratios and lower electronic mobility.^37,40^ Conversely, highly crystalline PGFs, such as those obtained *via* catalytic graphitization, show improved conductivity but present pore collapse or loss of ultra-micro pores needed for adsorption and ion transport.^7,14^ It can be emphasized that resolving this issue remains a challenge, which may not suitable across systems.

The inconsistencies in pore-size characterization, defect quantification, and graphitization indexing differ across measurement techniques and can lead to inconsistent interpretations across studies. For example, Kr adsorption and GCMC simulations provide Å-level accuracy for micropore distributions, but they are not yet standard in non-nuclear graphite research.^36^ Similarly, Raman *I*_D_/*I*_G_ ratios vary with laser wavelength and cannot always distinguish between edge defects and in-plane vacancies. PDF analysis, while powerful, remains low capability of utilization due to instrumentation challenges.^14^

Raman spectroscopy clearly correlates disorder with pseudocapacitive enhancement in nanoporous carbons,^37^ but this conflict with the systems where improved crystallinity provides superior electron transport.^7^ Similarly, PDF analysis has revealed that many PGFs contain heterogeneous mixtures of amorphous and nanocrystalline domains, complicating predictions of performance based on XRD alone.^14^ Additionally, Kr adsorption has recently emerged as a sensitive probe for edge-plane density. However, few studies employ this technique despite its utility in correlating defect sites with catalytic performance.^36^ These studies imply the gaps in comparison across PGF systems.

Although structural analysis tools (N_2_ sorption, Raman, XRD, PDF, Kr adsorption) have become highly useful, there is no unified quantitative framework linking hierarchical porosity, graphitic domain size, edge-plane density, and electronic structure. For example, PDF analysis reveals coexistence of amorphous and nanocrystalline carbon regions,^14^ Raman *I*_D_/*I*_G_ correlations vary by excitation wavelength,^37^ and Kr adsorption probes edge-plane density^36^ but it is hardly used outside nuclear graphite research. Standardized measurement is essential for future PGF design.

## 4. Properties

### 4.1. Electrical conductivity

**Fig. 4:**
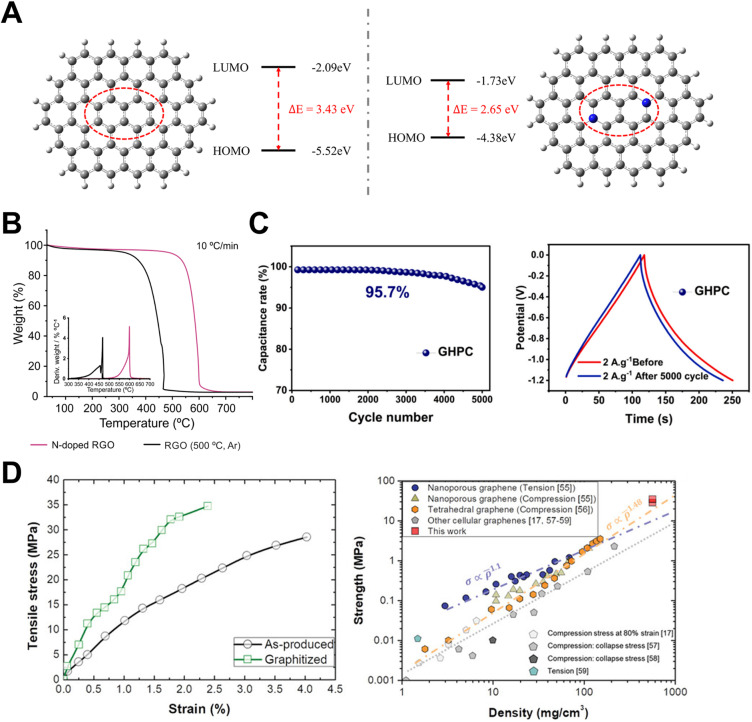
(A) Density functional calculations of energy gaps in pyridinic N-doped and undoped carbon materials.^41^ Reuse permission from Wang *et al.* (2025)^41^ by The Royal Society of Chemistry, under a Creative Commons Attribution 4.0 International License (https://creativecommons.org/licenses/by/4.0/). (B) TGA curves of the RGO and N-doped RGO.^42^ Reuse permission from Martincic *et al.* (2024)^42^ by MDPI, under a Creative Commons Attribution 4.0 International License (https://creativecommons.org/licenses/by/4.0/). (C) Cyclic stability of the GHPC electrode.^30^ Reuse permission from Guye *et al.* (2025)^30^ by Elsevier. (D) Stress–strain curves and strength *versus* density of porous graphitic carbon materials.^44^ Reuse permission from Polyakova *et al.* (2023)^44^ by MDPI, under a Creative Commons Attribution 4.0 International License (https://creativecommons.org/licenses/by/4.0/).

PGFs inherit the delocalized π-electron network of graphite, yet their conductivity is ultimately dictated by the continuity of sp^2^ domains and the density of charge-scattering defects.^40^ Four-probe measurements on a wood-waste-derived, N-doped multiporous carbon (N-GMPC) recently reached 9.4 S cm^−1^ while preserving a BET area of 1638 m^2^ g^−1^. Density-functional calculations attributed this synergy to pyridinic-N-induced band broadening, which lowered the carrier effective mass (Fig. 4A).^41^ Comparable conductivities (>10 S cm^−1^) were reported for hierarchical porous graphitic carbons synthesized by inside-out K/Fe activation, where atomically dispersed Fe catalyzed lattice ordering during pore generation.^34^ These studies overcome the classical porosity-conductivity tradeoff by demonstrating that controlled graphitization and heteroatom modulation can furnish bicontinuous electronic and ionic highways, which are prerequisites for high-rate electrochemical energy storage.

### 4.2. Thermal stability

The strong in-plane σ-bonds of graphitic carbon endow PGFs with exceptional resistance to thermally driven lattice degradation. Thermogravimetric analysis (TGA) of multilayer graphene and CNT analogs showed an oxidation onset (*T*_onset_) in air above 500 °C, rising toward 650 °C as the degree of graphitization increased (Fig. 4B).^42^ Hierarchical porous graphitic carbons fabricated by metallothermic reduction maintained 95% of initial capacitance when cycled at 150 °C in ionic-liquid electrolytes, confirming functional stability under harsh thermal loads.^43^ Potassium-catalyzed graphitization further lowers the energy cost of producing such heat-resilient carbons, enabling crystallite development below 1 000 °C without sacrificing porosity. This is a key sustainability advancement in large-scale syntheses.^32^

### 4.3. Chemical stability

Chemical inertness is critical when the interface with strongly acidic, alkaline, or oxidative. Self-heteroatom-doped porous carbons derived from *Mikania micrantha* retained >90% capacitance after 10 000 cycles in 1 M H_2_SO_4_, highlighting the role of graphitic domains in suppressing carbon corrosion.^45^ In alkaline media, bio-templated graphitic frameworks sustained 95.7% capacity over 5 000 cycles at 2 A g^−1^, with post-mortem X-ray photoelectron spectroscopy revealing minimal surface oxidation, again underscoring the chemical passivation imparted by extended sp^2^ conjugation (Fig. 4C).^30^ Molecular simulations of hydroxyl radical attack on graphitic edges indicated activation barriers of >1.5 eV, corroborating the experimental durability trends and suggesting that selective edge functionalization, rather than basal-plane degradation, governs long-term stability in electrochemical reactors.^46^

### 4.4. Mechanical strength

Although porosity usually compromises the mechanical integrity, PGFs leverage the high intrinsic modulus of graphene to achieve remarkable strength-to-weight ratios. Molecular-dynamics simulations predicted compressive strengths of up to 2 GPa at 70% strain for multilayer graphene networks, facilitated by out-of-plane buckling and interlayer friction that dissipate energy without catastrophic failure (Fig. 4D).^44^ Experimentally, MOF-derived porous graphitic carbon reinforced with residual metal nanonodes exhibited a Young's modulus of 34.8 MPa and recovered 95% of the original height after 1 000 compression cycles, demonstrating the super-elastic behavior desirable for flexible devices.^47^ These findings suggest that the strategic incorporation of graphitic struts and hierarchical cell walls can reconcile high surface areas with load-bearing capacities, thereby opening avenues for the development of structural energy-storage composites and thermally stable insulation foams.

Mechanical/thermal stabilities and conductivity are mutually dependent. Therefore, it is difficult to optimize simultaneously. Insights from examples, such as conductive N-doped carbons (>9 S cm^−1^)^41^ and solid nanocellular graphene architectures,^47^ underscore the balance between defect density, graphitic order, and porosity. The challenge is that multi-property optimization (porosity, conductivity, mechanical stability) remains one of the field's most significant unsolved aspects. Collectively, the unique combination of high electrical conductivity, thermal/chemical robustness, and tunable mechanical resilience makes PGFs a versatile platform for next-generation electrochemical, catalytic, and structural applications.

## 5. Applications

### 5.1. Energy storage

**Fig. 5:**
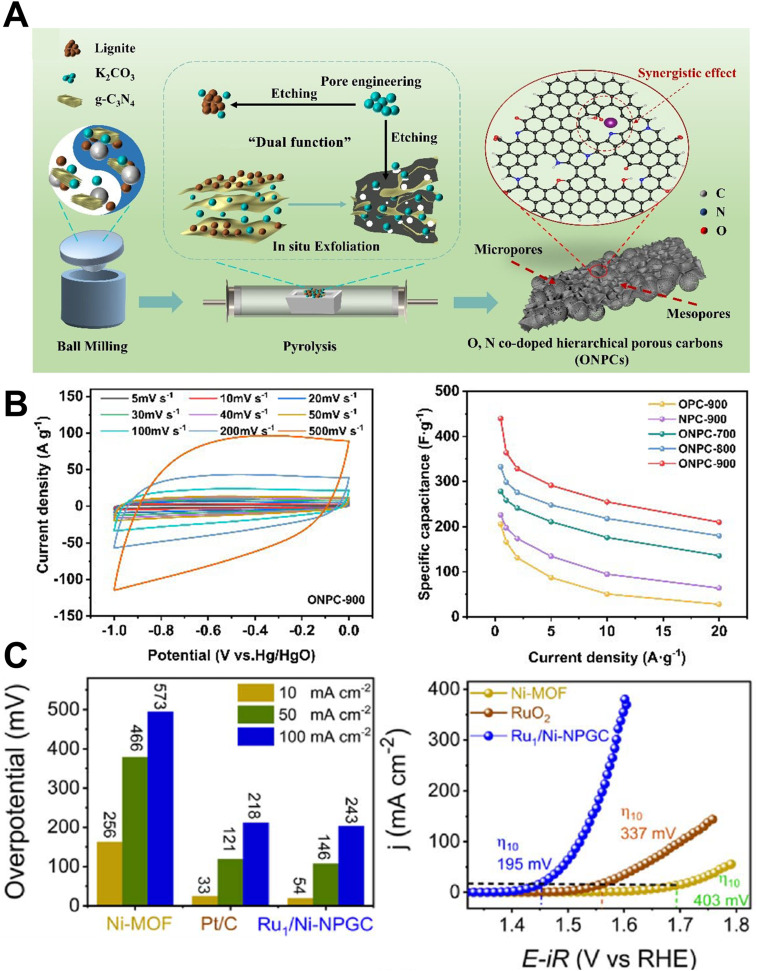
(A) Schematic of synergistically fabricated ONPC materials.^33^. (B) CV plots of ONPC-900 measured at various scans (5–500 mV s^−1^) and SC comparison of ONPC materials measured at different current densities.^33^ Reuse permission from Liu *et al.* (2025)^33^ by OAE Publishing Inc., under a Creative Commons Attribution 4.0 International License (https://creativecommons.org/licenses/by/4.0/). (C) HER performance of Ru1/Ni-NPGC catalysts and *iR*-corrected OER polarization curves (scan rate 1 mV s^−1^).^13^ Reuse permission from Mishra *et al.* (2025)^13^ by The Royal Society of Chemistry.

The integration of high surface area, and hierarchical porosity with extended sp^2^ conjugation continues to elevate the performance of PGFs in electrochemical energy storage.^40^ For example, machine-learning (ML)-guided optimization of an O,N co-doped hierarchical PGF (ONPC-900) recently delivered a gravimetric capacitance of 440 F g^−1^ at 0.5 A g^−1^ and preserved 92% of that value after 10 000 cycles (Fig. 5A and B). Density-functional calculations ascribed the performance boost to synergistic ion adsorption at the pyrrolic-N and -COOH sites embedded in the graphitic walls, demonstrating the synergistic contribution of O,N surface functionalities to ion-accessible adsorption sites and conductivity.^33^ Similarly, high-surface-area hierarchical PGFs derived from conjugated polymer frameworks, with high SSA (approximately 4073 m^2^ g^−1^) deliver rapid charge–discharge behavior without the diffusion limitations typically observed in activated carbons.^7^ Complementary “inside-out” K/Fe activation-graphitization has demonstrated that conductive graphitic backbones can coexist with 3 000 m^2^ g^−1^ of accessible surface, yielding 322 F g^−1^ and 101% capacitance retention over 15 000 cycles in symmetric supercapacitors.^34^ These studies collectively show that heteroatom chemistry and catalytic graphitization can reconcile the historic trade-off between porosity and electronic conductivity, enabling PGF electrodes that operate at >10 A g^−1^ without severe IR drop, which attributes now being translated into Zn-ion hybrid and Li–S full-cell architectures. It demonstrates PGFs as one of the few materials classes that can provide both rate and stability requirements for next-generation supercapacitors.^34,48^

### 5.2. Catalysis

In electrocatalysis, PGFs provide tunable defect topologies and high electronic mobility that effectively stabilize atomically dispersed active sites.^13^ The catalytic performance of PGFs depends on the interplay between the edge-plane defects (active sites) and graphitic continuity (electron transport). A pore-edge graphitic-nitride framework prepared *via* SiO_2_-protected templating exhibits a half-wave potential of 0.842 V for the oxygen-reduction reaction and sustains 779 mA h g_Zn_^−1^ in liquid Zn–air batteries, outperforming numerous metal- N- C benchmarks. The authors attributed this activity to the vacancy-enriched N at the pore rims coupled with hierarchical ion pathways.^49^ Extending this paradigm, Ru–N_4_ single atoms anchored on Ni-MOF-derived PGF achieve overpotentials of only 54 mV (HER) and 195 mV (OER) at 10 mA cm^−2^ (Fig. 5C) across the full pH window. They are competitive with noble metal benchmarks.^49^ This observation underscores the mechanism by which atomically dispersed metals are stabilized by the strong metal–support interactions within graphitic lattices.^13^ These results highlight the potential of PGFs as tunable corrosion-resistant scaffolds for both electrocatalytic redox reactions and thermocatalytic transformations.

### 5.3. Gas storage and separation

**Fig. 6:**
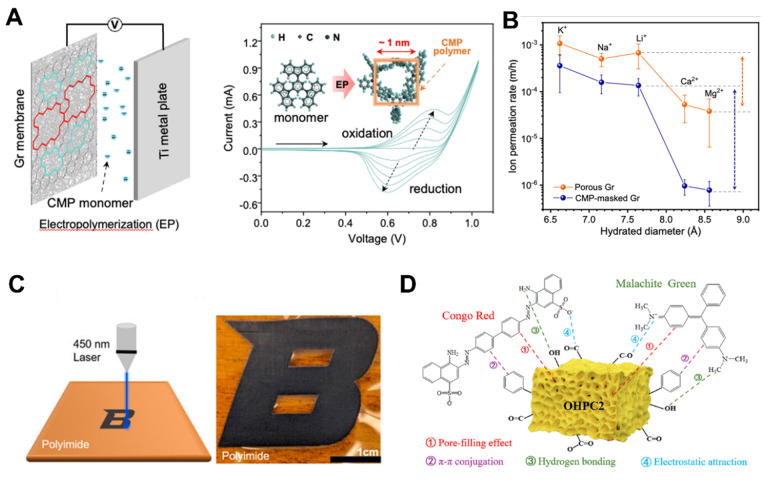
(A) Schematic of the electrochemical repair device and cyclic voltammetry profiles of the electrochemical repair process.^51^. (B) Ion–ion separation performance of CMP-masked porous graphene membranes.^51^ Reuse permission from Zhou *et al.* (2024)^51^ by Springer Nature, under a Creative Commons Attribution 4.0 International License (https://creativecommons.org/licenses/by/4.0/). (C) Schematic of LIG fabrication and optical image of the patterned LIG.^52^ Reuse permission from Francis *et al.* (2024)^52^ by Frontiers Media S.A., under a Creative Commons Attribution 4.0 International License (https://creativecommons.org/licenses/by/4.0/). (D) Schematic of the possible mechanisms of CR and MG adsorption by OHPC2.^53^ Reuse permission from Zhang *et al.* (2025)^53^ by Elsevier.

The ultra-micro porous regime (<0.7 nm) accessible in PGFs affords binding enthalpies well-matched to H_2_ and CO_2_ physisorption. A review of adsorption-based H_2_ storage reports that graphitic carbons with pore volumes enriched below 0.6 nm routinely surpass 6 wt% uptake at 77 K and 100 bar, with sp^2^ content emerging as a key predictor of isosteric heat.^50^ For separations, defect-engineered monolayer graphene repaired by a 10 nm conjugated microporous polymer masks non-selective pores and achieves Li^+^/Mg^2+^ selectivity of 300 alongside high flux, demonstrating Å-scale sieving combined with mechanical integrity (Fig. 6A and B).^51^ Recent breakthroughs in Å-precision porosity illustrate the growing potential of PGFs in gas purification. Carbon molecular sieves incorporating single Zn atoms and tightly confined ultra-micro pores demonstrate H_2_/CO_2_ selectivity values reaching 130, and H_2_/CH_4_ selectivity as high as 2900. These performance metrics that exceed the Robeson bounds for polymer membranes.^9^ These results come from finely-tuned pore in the 2.1–4.0 Å range. They are regulated by carbonization temperature and metal-assisted carbon rearrangements. The graphitic backbone provides both mechanical chemical stability, enabling PGF-derived membranes to operate under rough thermal and pressure that challenge traditional polymer systems. These advances have enabled PGF membranes to meet the simultaneous demands of permeability, selectivity, and stability required for hydrogen purification and post-combustion CO_2_ capture.

### 5.4. Sensors

Porous graphitic architectures also underpin next-generation chemiresistive platforms. Laser-induced graphene (LIG) fabricated on polyimide *via* 450 nm photothermal writing yields a defect-rich (Fig. 6C), 3D graphitic foam whose resistance changes by up to 5% in response to sub-ppm volatile organic compounds at room temperature. The porous morphology and nanometer-scale crystallites provided abundant adsorption sites while maintaining flexibility over 100 bending cycles.^52^ The low-cost and patternable nature of LIG demonstrate how PGF principles can be leveraged for scalable environmental monitoring and wearable analytics.

### 5.5. Adsorption

Finally, the combination of hierarchical porosity and π-rich basal planes endows PGFs with exceptional sorptive capacities for aqueous contaminants. An oxidized hierarchical porous carbon featuring vesicule-like ultrathin graphitic walls exhibits Langmuir capacities of 2.729 mg g^−1^ for Congo red and 1.697 mg g^−1^ for malachite green, with regeneration efficiencies above 90% over five cycles. These capacities exceed those of typical activated carbons, due to the combined mechanistic contributions of π–π stacking, pore filling, surface hydrogen bonding, and favorable electrostatic interactions within ultrathin graphene-like domains (Fig. 6D).^53^ This advantageous performance, achieved without metal additives, positions PGFs as sustainable adsorbents for dye-rich industrial effluents and emerging micropollutants.

### 5.6. Practical limitations and perspectives

Supercapacitor studies using high surface-area PGFs often demonstrate excellent cycling stability of electrodes at small laboratory scale but do not report volumetric performance, electrolyte compatibility, or mechanical integration, which factors crucial for device-level use.^34,48^ Although single-atom PGF electrocatalysts such as RuN_4_ show excellent HER/OER kinetics,^13^ long-term tolerance and large-scale electrode integration remain elusive. Besides, their stability under industrial electrolyte impurities, agglomeration resistance, and mass transport under realistic flow conditions are not yet adequately explored.^13^ Membrane applications (H_2_/CO_2_ separation) using ultra-micro porous PGFs^9^ shows superior selectivity but meet the challenges in mechanical stability, defects control, and trade-offs between permeability and long-term changes in selectivity. These issues limit of the technological applications of PGF-based devices.

For energy storage, O,N-co-doped hierarchical PGFs^33^ achieve high gravimetric capacitance but lack data on volumetric performance, electrode densification, and industrial electrolyte compatibility. In catalysis, Ru–N_4_ PGFs^30^ show outstanding HER/OER activity, however, long-term stability against metal migration remains less understood. For gas separation, single-atom Zn-embedded molecular sieve carbons^9^ demonstrate remarkable H_2_/CO_2_ selectivity, but still face mechanical limitations in membrane-scale fabrication. For sensing, laser-induced graphitic foams^52^ show high sensitivity but require improvements in signal and environmental stabilities. It can be clearly stated that scaling from gram-scale performances to device-level deployment is the most critical next step. These cases illustrate method limitations that remain unresolved.

Collectively, these application domains show that rational control over the pore architecture, graphitic order, and heteroatom chemistry transforms PGFs from structural curiosities into multifunctional workhorses spanning energy, environment, and sensing technologies (Table 2).

**Table 2:** Comparative performance metrics of PGFs in various applications

Applications	Representative PGF	Key structural features	Performance metrics	Ref.
Supercapacitors	ONPC-900	Hierarchical micro–mesoporosity; O,N co-doping	440 F g^−1^ at 0.5 A g^−1^; 92% retention after 10 000 cycles	33
Supercapacitors	HPG carbon	Crosslinked PANi-derived 3D conjugated framework	BET ∼4073 m^2^ g^−1^; high-rate stability	7
Electrocatalysis (HER/OER)	Ru–N_4_/Ni-MOF-PGF	Atomically dispersed Ru–N_4_ sites; graphitic channels	54 mV (HER) and 195 mV (OER) at 10 mA cm^−2^	13
ORR catalysis	Pore-edge graphitic nitride PGF	Vacancy-enriched N, hierarchical channels	Half-wave potential: 0.842 V; Zn–air discharge: 779 mA h g_Zn_^−1^	49
H_2_/CO_2_ separation	Zn-embedded CMS (PGF-like)	Å-precision ultra-micro pores *via* controlled carbonization	Selectivity: H_2_/CO_2_ = 130; H_2_/CH_4_ = 2900	9
Gas adsorption (pollutants)	OHPC2	Vesicle-like ultrathin graphitic walls	*q*_max_ = 2729 mg g^−1^ (Congo red); 1697 mg g^−1^ (MG)	53
Mechanical stability	MOF-derived graphitic nanocellular carbon	Interconnected graphitic struts; metal nanonodes	Young's modulus: 34.8 MPa; 95% height recovery after 1000 cycles	47
Thermal stability	Hierarchical graphitic carbon	High graphitization index	Capacitance stability at 150 °C over 10 000 cycles	43

## 6. Outlook and future perspectives

### 6.1. Advanced synthesis techniques

The next decade could shift from slow equilibrium-controlled pyrolysis to kinetically driven data-assisted manufacturing routes for PGFs. Ultrafast flash-Joule heating (FJH) already converts diverse solid wastes or graphene-oxide aerogels into milligram-to-kilogram batches of 3D “flash graphene” within <1 s, reaching peak temperatures >3 000 K and specific surface areas above 1 000 m^2^ g^−1^ while consuming only 2–3 kWh kg^−1^ of the product.^35,54^ Scientific machine-learning models trained on FJH process variables can predict graphene yields with *R*^2^ ≈ 0.8, functioning as closed-loop reactors that optimize porosity and graphitic order in real time.^54^ In parallel, molten-salt media such as LiCl/KCl or ZnCl_2_ not only act as high-temperature solvents but also template nanoscale voids and scavenge heteroatoms. A recent molten-salt-assisted route produced Ru- single-atom/porous-N-graphitic carbons with metal loadings of up to 4.3 wt% and turnover frequencies rivaling those of Pt for water electrolysis.^19^ Additive manufacturing closes this gap in geometric properties. Inverse-designed electrodes fabricated by direct-ink-writing of graphene aerogel lattices lowered power losses in flow reactors by 16% relative to homogeneous porosity controls, proving that micron-resolved porosity gradients can be printed and pyrolyzed without sacrificing electrical consecutivenes.^55^ Collectively, the convergence of FJH, molten-salt chemistry, and 3-D printing promises kilogram-scale production of architected PGFs with programmable pore topology/crystallinity.

### 6.2. Enhanced functionalization

Beyond skeleton engineering, the future performance of devices will be based on precise electronic and chemical tailoring of the graphitic backbone. Single-atom metallurgy is a rapidly developing field of research. For instance, Ru–N_4_ moieties stabilized on Ni-MOF-derived PGFs drive pH-universal overall water splitting at 10 mA cm^−2^ with overpotentials of only 54 mV (HER) and 195 mV (OER), benefiting from strong metal–support interactions within nitrogen-coordinated graphene cavities.^13^ Additionally, post-synthetic covalent chemistry offers a complementary avenue. Advances in diazonium, click, and Pictet–Spengler reactions have enabled nanometer-resolved grafting of redox mediators or ionic anchors without collapsing the pore network, as exemplified by a one-pot Pictet–Spengler polymerization that installed proton-conducting benzimidazole rings throughout a mesoporous graphitic scaffold.^56^ In addition, edge-selective ball-milling and plasma routes deliver kilogram-scale heteroatom doping with controllable N, P, or S speciation, as summarized in a study on sp^2^-carbon functionalization.^57^ A promising trend is the development of a toolbox that couples atomic precision with macroscopic throughput, allowing catalytic, sensing, or sorptive sites to be defined independently of bulk conductivity.

### 6.3. Emerging applications

The structural and chemical versatility unlocked above provides a catalytic application space that extends well beyond classical electrochemistry. In electromagnetic-interference (EMI) shielding, hierarchical CNT/graphitic-carbon foams with engineered pore/graphite synergies already deliver >80 dB X-band attenuation at thicknesses of only 160 µm, an absorption-dominated mechanism that is attractive for lightweight aerospace electronics.^58^ Gas-hydrate energy storage has also been proposed. Ultralight graphene aerogels act as fixed beds that nucleate methane hydrates inside 50–250 µm pores, raising volumetric storage capacity to 146 V V^−1^ and mitigating expansion stresses that plague conventional pellets.^59^ Biomedical technology is another mechanically resilient hybrid aerogel composed of dual-scale fibers and graphitic nanoflakes that supports rapid tissue ingrowth and neovascularization, while retaining superelastic recovery (1.8 s) under compressive loads, opening prospects for minimally invasive soft-tissue scaffolds and bioelectronic interfaces.^60^ Finally, architectured PGF electrodes, printed by inverse design, support electrochemical flow reactors and redox-flow batteries with reduced pumping energy and enhanced kinetics, illustrating the emerging role of digital manufacturing in translating pore-scale insights into device-level efficiency gains.^55^

### 6.4. Perspectives

PGFs have progressed from conceptual hybrids to a coherent material family whose synthesis–structure–property relationships can now be articulated with increasing precision. Integrative routes that couple chemical activation with catalytic graphitization reconcile the long-standing porosity–conductivity trade-off, as exemplified by inside-out K/Fe strategies that deliver >300 F g^−1^ capacitance alongside 10 S cm^−1^ conductivity.^34^ Parallel molten-salt chemistries embed transient ionic media to template subnanometer voids and stabilize heteroatom-rich edge planes, yielding surface areas above 2500 m^2^ g^−1^ while endowing catalytic functionality.^61^ These advances confirm that hierarchical porosity, extended sp^2^ conjugation, and site-specific heteroatom doping are not mutually exclusive. Instead, they can be co-engineered to unlock superior electrochemical, catalytic, and adsorptive performances. Scalability and sustainability, which are historical bottlenecks for designer carbons, were addressed using equilibrium manufacturing paradigms. Flash-Joule heating now converts biomass or polymer waste into kilogram-scale flash graphene within seconds, cutting energy demand to <5 kWh kg^−1^ and reducing life-cycle CO_2_ emissions by approximately 86% relative to conventional graphitization.^31^ When integrated with automated control and data-driven process optimization, such ultrafast routes promise real-time tuning of pore topology and crystallinity, whereas molten-salt and additive-manufacturing techniques offer complementary pathways toward complex, application-specific architectures. Therefore, hybrid synthesis strategies (*e.g.*, molten-salt + CVD healing; FJH + activation) represent the most promising forward-looking path. Besides, real-time process control (AI/ML-guided synthesis, *in situ* monitoring) will soon redefine PGF reproducibility and scalability.^54,62^ Digital manufacturing and topology-optimized design will also unlock specific applications of PGFs for flow reactors, membranes, and energy devices.^55^ In addition, sustainable and circular-carbon PGFs prepared from biomass or waste streams^31,63,64^ will be increasingly essential. Collectively, these developments represent a potential route from laboratory gram-scale performances to the industrial production of architectured PGFs.

The functional breadth of the PGFs has expanded. Nitrogen-doped, bio-derived frameworks already rival state-of-the-art activated carbons in supercapacitors while leveraging agricultural waste streams.^63–65^ Absorption-dominated electromagnetic-interference shields achieve 80 dB effectiveness at ultralow areal densities, underscoring the utility of hierarchical graphitic foams in lightweight electronics.^66^ However, opportunities remain unexplored, such as Ångström-resolved membranes for ion-sieving, biocompatible aerogels for regenerative medicine, and redox-mediated flow electrodes for large-scale energy storage. These benefits are attributed to the unique confluence of conductivity, stability, and tunable porosity intrinsic to PGFs. Realizing these prospects requires standardized metrology for pore/defect quantification, spectroscopy to map coupled ion-electron transport, and multiscale modeling frameworks that bridge atomistic chemistry with device-level performance.

## 7. Conclusions

In summary, the synthesis, functionalization, and deployment of PGFs are co-developing toward a future in which atomic-to-device integration is common. Achieving this vision requires high-throughput, energy-saving fabrication with predictive modeling and application-specific surface chemistry, which is an interdisciplinary challenge that positions PGFs at the center of sustainable manufacturing, advanced catalysis, and next-generation multifunctional materials. The rapid development of advanced synthesis, precision functionalization, and emerging application spaces has made PGFs a promising option for next-generation carbon materials. Integration of sustainable manufacturing with atom-to-device design principles can be an effective strategy for developing multifunctional architectures to mitigate the challenges in clean energy, environmental remediation, and high-frequency electronics.

## Author contributions

Conceptualization, resources, writing, review, and editing: Minh Kim Nguyen, Ha Huu Do, Nguyen Tien Tran, and Young-Chul Lee.

## Conflicts of interest

The authors declare no conflicts of interest.

## Data Availability

No primary research results, software or code have been included and no new data were generated or analysed as part of this review.
